# Disaggregating the data: Diversity of COVID-19 stressors, discrimination, and mental health among Asian American communities

**DOI:** 10.3389/fpubh.2022.956076

**Published:** 2022-10-19

**Authors:** Sumie Okazaki, Christina Seowoo Lee, Aakriti Prasai, Doris F. Chang, Nari Yoo

**Affiliations:** ^1^Department of Applied Psychology, Steinhardt School of Culture, Education, and Human Development, New York University, New York, NY, United States; ^2^Silver School of Social Work, New York University, New York, NY, United States

**Keywords:** Asian Americans, data disaggregation, COVID-19 discrimination, mental health, distress, worry

## Abstract

Much of the public discourse as well as research regarding the negative impact of COVID-19-related anti-Asian discrimination has been conducted at the broad racial group level, yet data aggregation masks critical points of diversity among Asian Americans. We conducted an online survey of 620 Asian American adults in December 2020 and examined whether there were any demographic differences–including by ethnic subgroup and Chinese street race (being Chinese or being mistaken as Chinese)–in their experiences of COVID-19-related stress, direct and vicarious discrimination, and psychological outcomes. Our analyses found that younger age was correlated with higher reports of pandemic stress, discrimination, distress, and worry. Female and U.S.-born participants reported higher levels of pandemic stress and vicarious discrimination, but there were no gender or nativity differences in levels of direct discrimination. Being uninsured was also related to higher levels of pandemic stress, discrimination, and distress. East Asian Americans reported significantly lower frequencies of direct anti-Asian discrimination than did South Asian or Southeast Asian Americans, but the ethnic subgroups did not differ in their reports of vicarious discrimination. Of note, Chinese street race was not associated with either direct or vicarious discrimination. Separate hierarchical regression analyses for East Asian, South Asian, and Southeast Asian participants revealed that, regardless of ethnicity, racial discrimination significantly contributed to psychological distress and worry beyond the effects of pandemic stress. However, the three groups varied in the demographic indicators and COVID-19 stressors that were associated with psychological outcomes. Pandemic stress was more strongly associated with negative outcomes among South Asian Americans than East Asian and Southeast Asian Americans, and neither direct nor vicarious discrimination were associated with mental health among South Asian Americans. Direct discrimination, compared to vicarious discrimination, was a particularly robust predictor of both distress and worry among East Asian Americans. For Southeast Asian Americans, direct discrimination significantly predicted higher levels of distress, whereas vicarious discrimination predicted higher levels of worry. Vicarious discrimination was not significantly related to distress across ethnic subgroups. Results suggest that practitioners and policy makers would benefit from attending to these within-group differences in Asian Americans' experiences during the pandemic.

## Introduction

From the very early days of the COVID-19 pandemic, there has been an alarming rise in xenophobic scapegoating and racial harassment of Asians, as China and the Chinese were blamed for the spread of the COVID-19 virus. Researchers have already documented a marked rise in Sinophobic content on both mainstream and fringe social media (1). #StopAAPIHate was launched in mid-March 2020 in the U.S. as an online portal to collect reports of anti-Asian hate incidents, and by December 2021, it had received a total of 10,905 incident reports, including verbal harassment, shunning, and physical attacks against not just Chinese internationals and Chinese Americans but also Asian Americans from across the United States (2). In addition to first-hand experiences of victimization, a steady stream of news about violence against Asian and Asian American women and older adults, sometimes resulting in deaths, has added to the sense that anti-Asian racism unleashed in the early days of COVID-19 (3) is not only unremitting but is increasing over time.

Although much of the public discourse about anti-Asian hate and discrimination has referred to Asian Americans in the aggregate, and the efforts to combat anti-Asian hate have also used a more inclusive language that often included Pacific Islanders (such as in the frequently used social media hashtag #StopAAPIHate), the bulk of anti-Asian hate sentiments and assaults have been directed toward East Asian Americans and those who might be misidentified as Chinese (2). The designation of COVID-19 related discrimination and hate in pan-Asian terms implies that East, Southeast, and South Asian Americans have been impacted similarly by COVID-19 life stresses and discrimination; as such, the pan-Asian discourse overlooks the vast demographic differences that exist within Asian American communities and also erases historical and contemporary differences in racialization that may shape how they perceive and are impacted by racial discrimination that is experienced directly and vicariously. The present study sought to address the potential problems of treating Asian Americans as a monolithic population by disaggregating the data on pandemic stress, direct and vicarious racial discrimination, and mental health outcomes across various points of demographic diversity (e.g., ethnicity, nativity, age, gender, class) within Asian American population during the first year of the COVID-19 pandemic.

### Diversity of racial experiences among Asian Americans

Asian American is a racial category imposed on a population that is more diverse in national origins and immigration histories than other racial groups in the U.S., with no one nationality group predominating and a vast diversity of languages spoken (4). From the earliest days of U.S. history, Asian immigrants, refugees, and asylum seekers have arrived with distinct ethnic, cultural, linguistic, and religious identities but have been cast into a pan-Asian racial category (e.g., as “Orientals”) and seen as the embodiment of a foreign, dangerous, and inferior “East” in opposition to the “West.” As the concept of race in the United States has been used–historically and contemporaneously–to support racism and to maintain racial hierarchy (5), racialization as Asian Americans has served to erase critical points of diversity. However, it is critical to recognize that different Asian groups have faced divergent forms of racialization and racism. Asian Americanist scholars e.g., (6, 7) have argued that non-White immigrants are either ideologically Whitened or ideologically Blackened according to their perceived socioeconomic status, and thus the failure to conform to the model minority image has resulted in the ideological Blackening of Southeast Asian communities. For example, Southeast Asian (e.g., Vietnamese, Lao, Cambodian) American youth identified with Black youth, with whom they shared a class background (7). Another study found that while South Asians were generally likely to label people from the Indian subcontinent (e.g., Pakistan, India) as Asian, all other groups, including Black, Latino, White, and other Asian Americans were significantly less likely to label people from the Indian subcontinent as Asian (8).

Even within the broad Southeast Asian grouping, which includes those who trace their origins to nations in the Indochinese peninsula (e.g., Thailand, Vietnam, Cambodia, Laos) as well as those from the islands and archipelagos (e.g., the Philippines, Malaysia, Indonesia), vast differences exist. For example, because of the history of imperialism and colonization from the United States and Spain, Filipino Americans are largely Catholic English speakers (9) and thus racialized differently from Vietnamese, Cambodian, and other mainland Southeast Asians. Chutuape (10) found that Filipino American youth in her study resisted a panethnic Asian American identity because they perceived those characteristics and assumptions about Asian Americans applied only to East Asians (e.g., Chinese, Korean, Japanese) (10). Many Southeast Asians (Vietnamese, Cambodian, Lao, and Hmong) entered the U.S. as refugees escaping war and violence following the Vietnam War, who–with the exception of first-wave Vietnamese immigrants from more educated and affluent classes in Vietnam–have experienced more barriers and challenges in their educational and occupational attainment than East Asians (11).

South Asian Americans are frequently excluded from Asian American discourse (12), and many South and Southeast Asian Americans identify as being Brown Asian Americans whose racial experiences are distinct from those of East Asian Americans (13). For example, after the terrorist attacks on September 11, 2001, there was a sharp rise in violence and hate crimes toward those who are Muslim and/or perceived to be Muslim. Through this process, many Americans who trace their heritage to the Middle East, North Africa, and South Asia were subject to Islamophobia regardless of faith. These attacks on South Asian Americans continue to this day, with Sikh American and visibly Muslim Americans being especially targeted (14, 15). Studies prior to the COVID-19 pandemic found South Asians to be especially vulnerable to forms of institutional discrimination and microaggressions, even when compared to other Asian American subgroups (16, 17). Other studies had also found that discrimination against South Asian Americans were associated with an increase in depressive symptomology following incidents of perceived discrimination, indicating the negative impact of discrimination on mental health (18, 19).

East Asian Americans, who are seen as the prototypical Asian Americans, have been racially vilified as the “yellow peril” throughout history of European and U.S. entanglements with East Asia, gaining force in the late 19th and early 20th century. The yellow peril racial trope cast the “yellow race” as posing economic, political, military, and cultural threats to the White race and led to a series of discriminatory and exclusion laws as well as the incarceration of Japanese Americans during the World War II (20). The yellow peril discourse casting East Asians (and in particular, Chinese, Chinese Americans, and other Asian Americans who are misidentified as Chinese) as a foreign threat has resurfaced in Sinophobic infectious disease narratives (e.g., related to SARS and COVID-19) (21).

### COVID-19 stress, discrimination, and distress

Despite the divergent historical and contemporary racialization experiences of various Asian American groups, much of the U.S. discourse around COVID-19-related stress and anti-Asian racism has referred to Asian Americans in the aggregate, and social science research has also followed suit. Although much of the public health data on the COVID-19 disease burden for Asian Americans are presented in the aggregate, there are compelling sociodemographic risk factors that vary widely across Asian ethnic groups (e.g., risk of exposure due to proportion of front-line workers, number of multigenerational households, barriers to healthcare, pre-existing health conditions, etc.) (22). A disaggregated analysis of COVID-19 case positivity, hospitalization, and deaths among Asian Americans in public hospital system in New York City found that South Asian patients had the highest rates of case positivity and hospitalization within Asian Americans, whereas Chinese Americans had the highest mortality rate of all racial and ethnic groups (23). Yet, the bulk of available research on Asian Americans' mental health during the pandemic has not included an assessment of life stresses associated with the COVID-19 disease.

There have been a number of studies documenting the relationship between anti-Asian racial discrimination and mental health outcomes in Asian American communities in the aggregate. For example, in an analysis of data collected in April to June 2020, Hahm et al. (24) found that 68% of 211 Asian and Asian American young adults (ages 18–30) reported that they or a member of their family experienced COVID-19 related discrimination, with ~15% reporting verbal and/or physical assaults. In the same sample, these discrimination experiences were associated with increases in posttraumatic stress disorder symptoms (24). Several other studies have also pointed to a significant link between Asian Americans' COVID-19 related racism and discrimination experiences and a host of negative mental health outcomes including symptoms of depression, post-traumatic stress, and anxiety (25–28).

Taken together, the association between anti-Asian discrimination and psychological distress appears to be well-established. Although many of the studies to date did not disaggregate their analyses by Asian ethnicities, some studies have sought to understand the link between discrimination and mental health separately or comparatively within Asian American ethnicities. For example, in an analysis of 245 Asian American adult surveyed, Woo and Jun (28) found that the effects of racial discrimination on depressive symptoms did not differ between Chinese Americans and other Asian subgroups (28). A study of 636 Chinese and South Asian American adults residing in Chicago found that depressive symptoms were significantly higher among those who were surveyed after the start of the COVID-19 pandemic than those who were surveyed prior to the start of the pandemic. Specifically, they found that South Asian American, men, and younger adults surveyed after the start of the pandemic reported more depressive symptoms compared to East Asian Americans, women, and older adults (29). Hyunh et al. (26) found in their sample of 380 East and Southeast Asian American adults in Ohio that their respondents reported an increase in direct and indirect racial discrimination during the pandemic compared to the time before the pandemic, and racial discrimination during the pandemic was associated with higher anxiety and depression (26). Notably, although there were no differences between East Asian and Southeast Asian Americans in the level of COVID-19 related racial discrimination, those whose ethnic identity was not Chinese reported higher depression and anxiety than those who identified as Chinese.

A handful of studies have also focused solely on Chinese Americans, who have been more immediately targeted and blamed by politicians and media figures (e.g., “China virus,” “Kung flu”) in the public discourse. For example, in a study of 543 Chinese American parents and 230 of their children between ages 10 and 18 early in the pandemic, Cheah et al. (30) found that nearly half reported being targeted by anti-Asian discrimination and Sinophobia, and those who reported being targeted also had worse mental health outcomes (30). A study with 198 Chinese American college students found that perceived xenophobia and anxiety were higher in the early days of the pandemic compared to before (31). In a survey of 184 Chinese American adults, Litam and Oh (32) found that COVID-19-related racial discrimination was associated with increased depressive symptoms and decreased life satisfaction; moreover, middle-aged men reported more discrimination and depression than younger or older men or women (32). In a study of 342 East Asian and East Asian American adults who were surveyed at three timepoints 2 weeks apart, (33), COVID-19 racism at each time point predicted later psychological distress. These disaggregated studies of COVID-19 related discrimination and mental health suggest that there are divergent experiences among Asian Americans depending on one's social location.

## The present study

Given the divergent historical and contemporary racialization of various Asian American groups, our primary aim was to disaggregate the population into meaningful ethnic subgroupings (East Asian American, Southeast Asian American, and South Asian) and to examine potential differences in the levels of COVID-19 stressful life events, direct and vicarious racial discrimination, and mental health outcomes. We based our exploratory study of relationships between pandemic stress, anti-Asian racism, and mental health on Harrell's (34) multidimensional conceptualization of racism-related stress (34), which integrates the literature on racism, stress processes, and mental health, as well as on Meyer's (35) minority stress theory, which posits that societal stigma, prejudice, and discrimination create a hostile environment that leads to mental health problems. Both theories predict that the stress associated with being a minority (being subjected to direct and vicarious anti-Asian prejudice and hate) are associated with negative wellbeing, above and beyond the effects of general stress (pandemic stress).

In addition to the ethnic subgrouping, we also examined whether being Chinese and/or having been mistaken for Chinese by strangers (“Chinese street race”) may be associated with experiences with racial discrimination. A previous study of street race (i.e., one's belief about how strangers on the street perceive your race/ethnicity) has found that Latinx who are racialized on the street as Black or Arab/Middle Eastern were more likely to have experienced racial discrimination (36). Moreover, because prior studies of Asian Americans' mental health during the pandemic have not paid adequate attention to other critical dimensions of heterogeneity within the population that may reflect structural inequalities (37), we examined the demographic correlates of mental health outcomes separately for Asian ethnic subgroups.

Specifically, we aimed to answer the following research questions: (1) To what extent did Asian American adults report various COVID-19-related stressors (namely, pandemic stress, direct anti-Asian discrimination, vicarious anti-Asian discrimination) during the early months of the pandemic, and how did these differ by major demographic characteristics? (2) To what extent did Asian American adults report experiencing distress and worry during the early months of the pandemic, and how did these differ by major demographic characteristics? (3) Were higher levels of direct and vicarious discrimination associated with worse psychological wellbeing (i.e., distress and worry) above and beyond the effects of pandemic-related life stress? And how did these associations differ by Asian ethnic subgroup?

## Methods

### Survey administration

Qualtrics, a commercial survey company, was contracted to collect online survey data from their research panel of respondents who had signed up to take online surveys in exchange for incentives (e.g., cash, airline miles, gift cards). The recruitment targeted potential respondents using the following eligibility criteria: (1) identify as Asian American, (2) are 18 years old or older, (3) able to respond to questions in English, and (4) had resided continuously in the United States between March 2020 and the date of the survey (in December 2020). Qualtrics used attention checks (i.e., embedding questions that instruct respondents to mark a specific response) and speeding checks (i.e., monitoring the duration of respondent survey engagement) as data quality checks. The final pool included responses from 689 Asian American adult respondents. Because of our research questions about broad Asian ethnic subgroups (i.e., East Asian, Southeast Asian, South Asian), we selected only those who did not indicate multi-ethnic or multi-racial heritage, which resulted in the sample of 620 for the present analyses.

### Demographic characteristics

Demographic characteristics of the total sample and each ethnic subgroup are reported in Table 1. Participants were 620 Asian American adults (61.29% female) whose age ranged from 18 to 80 years (*M* = 40.6, *SD* = 15.7). Our sample consisted of 340 (54.84%) East Asian, 153 (24.68%) Southeast Asian, and 127 (20.48%) South Asian participants. The largest ethnic subgroups were Chinese (*n* = 209; 33.71%), Indian (*n* = 81; 13.06%), Filipino (*n* = 70; 11.29%), Japanese (*n* = 59; 9.52%), Korean (*n* = 47, 7.58%), and Pakistani (*n* = 23; 3.71%). Participants represented 43 states spanning diverse geographic regions including the West (40.97%), Northeast (21.94%), South (25.97%), and Midwest (11.13%). More than half (55.00%) of the sample was born outside of the United States, and the average length of U.S. residence among foreign born participants was 25.5 years (SD = 15.0). The majority of participants had received some college education or more (80.97%) and reported their relationship status as either single (39.90%) or married (44.43%). Our sample was predominantly heterosexual (90.95%), with the remainder identifying as gay/lesbian (3.07%), bisexual/pansexual (4.85%), and other (1.13%). In terms of political affiliation, 41.13% of participants identified as liberal, 36.45% identified as neither liberal nor conservative, and 22.42% identified as conservative. Approximately 40% of participants reported not having a religious affiliation, with the majority of the rest identifying as Protestant (18.23%), Catholic (11.61%), Buddhist (10.00%), Hindu (8.87%), and Muslim (6.29%).

**Table 1:** Sample characteristics by ethnic subgroup.

	**Total (*n* = 620)**	**East Asian (*n* = 340)**	**South Asian (*n* = 127)**	**Southeast Asian (*n* = 153)**	**χ^2^/*F***
**Variables**	***n* (%)**	***n* (%)**	***n* (%)**	***n* (%)**	
Age, *M* (*SD*)	40.63 (15.70)	44.23 (16.04)	36.78 (15.71)	35.83 (12.69)	*F* = 21.18***
Gender					
Male	240 (38.71)	141 (41.47)	47 (37.01)	52 (33.99)	χ^2^ = 2.69
Female	380 (61.29)	199 (58.53)	80 (62.99)	101 (66.01)	
Nativity					
U.S. born	279 (45.00)	134 (39.41)	69 (54.33)	76 (49.67)	χ^2^ = 10.11**
Foreign born	341 (55.00)	206 (60.59)	58 (45.67)	77 (50.33)	
Education					
Less than college	118 (19.03)	56 (16.47)	25 (19.69)	37 (24.18)	χ^2^ = 4.12
Some college or more	502 (80.97)	284 (83.53)	102 (80.31)	116 (75.82)	
Health insurance					
Uninsured	57 (9.56)	22 (6.67)	16 (13.33)	19 (13.01)	χ^2^ = 7.25
Public insurance	137 (22.99)	77 (23.33)	27 (22.50)	33 (22.6)	
Private insurance	402 (67.45)	231 (70)	77 (64.17)	94 (64.38)	
Geographic region					
Northeast	137 (21.92)	79 (23.10)	45 (35.16)	13 (8.39)	χ^2^ = 73.37***
West	255 (40.8)	166 (48.54)	17 (13.28)	72 (46.45)	
South	164 (26.24)	63 (18.42)	52 (40.63)	49 (31.61)	
Midwest	69 (11.04)	34 (9.94)	14 (10.94)	21 (13.55)	
Street race					
Chinese or mistaken					
as Chinese	366 (58.56)	290 (84.8)	4 (3.13)	72 (46.45)	χ^2^ = 268.22***
Neither Chinese nor					
mistaken as Chinese	259 (41.44)	52 (15.2)	124 (96.88)	83 (53.55)	

**p < 0.01, ***p < 0.001.

### Measures

#### Pandemic stress

Pandemic stress was measured using the Holmes and Rahe Stress Inventory (38–40). Participants were shown a list of 43 life events that could have happened that would have resulted in a change in their lives, and they were asked to indicate whether any of the events had happened since January 2020. Each event is associated with a numerical index of how challenging it is to adapt to the changes caused by that particular event. These life events ranged from death of a spouse (100), death of a close family member (63), change in financial state (38), major change in living condition (25), to minor violations of the law such as traffic tickets (11). The scale is scored as the sum of life change units accrued during the specified period, with the theoretical range from 0 to 1,000. The Holmes and Rahe Stress Inventory has been shown to be positively correlated with other measures of stress in the general U.S. population [e.g., Global Inventory of Stress Scale; (41)] and demonstrated good test-retest reliability (*r* = 0.82) over a two-week period (42). It has also been found to be valid across different U.S. ethnic populations (African American, Mexican American, White American) as well as in some overseas populations including in Japan and Taiwan (43) and Taiwan (40). We divided the sum score by 10 to facilitate the interpretation of regression coefficients.

#### COVID-19 related racial discrimination

Experiences of discrimination were assessed using a modified version of the COVID-19 Related Racial Discrimination Scale (30), which assessed four types of racial discrimination experiences that may have affected Chinese Americans during the pandemic: direct in-person, direct online, vicarious in-person, and vicarious online. Following Zong et al. (44), the present study collapsed these subscales to understand direct discrimination either online or in-person and vicarious discrimination either online or in person. For each item in these scales, participants indicated the frequency at which they experienced the event since the start of the pandemic. To account for the time that has passed since spring 2020, we revised the original 6-point Likert scale ranging from 1 (never) to 6 (every day) to a 5-point Likert scale ranging from 1 (never) to 5 (very frequently, 11+ times). The summary scores were calculated as the mean value across the items for each subscale. The internal consistency (Cronbach's alpha or α hereafter) of the combined direct discrimination subscale in our sample was excellent for the overall sample (α = 0.95) and across ethnic subgroups (α = 0.94, 0.96, and 0.95 for East Asians, South Asians, and Southeast Asians, respectively). The vicarious discrimination subscale also has an excellent internal consistency for the overall sample (α = 0.93) and across ethnic subgroups (α = 0.94, 0.92, and 0.92 for East Asians, South Asians, and Southeast Asians, respectively).

#### Psychological distress

Psychological distress was measured using the 10-item version of the Kessler Psychological Distress Scale (K10) (45), a simple measure of non-specific psychological distress that has been used with racial/ethnic minorities including Asian Americans (46). Research has supported a two-factor structure of this scale, including Depression and Anxiety, across various samples including Chinese adults (47). Participants responded to a series of 10 questions about how often they felt emotional states including feeling tired, hopeless, nervous, or depressed during the last 30 days, with response options including 1 (none of the time), 2 (a little of the time), 3 (some of the time), 4 (most of the time), and 5 (all of the time). Total scores, computed as the sum of scores across the 10 items, range from 10 to 50, with scores from 20 to 24 indicating a “mild mental disorder,” scores from 25 to 29 indicating a “moderate mental disorder,” and scores above 30 indicating a “severe mental disorder.” The validity of these cutoffs has been supported by their ability to discriminate between individuals with mental illness vs. those without (45, 48). The internal consistency of this subscale in our sample was excellent for the overall sample (α = 0.96) and across ethnic subgroups (α = 0.95, 0.97, and 0.96 for East Asians, South Asians, and Southeast Asians, respectively).

#### Worry

Worry was measured using the brief version of the Penn State Worry Questionnaire (49). This scale consisted of five items including “Many situations make me worry,” “I know I should not worry about things, but I just cannot help it,” and “I noticed that I have been worrying about things.” Participants were asked to indicate if those statements were typical of them, with options ranging from 1 (not at all typical of me) to 5 (very typical of me), and total scores were calculated as the sum of the five items, with a theoretical range between 5 and 25. Higher total scores indicated higher levels of worry, with a cut-off score of 15 or greater suggesting clinical level of worry (49). While the brief version of the PSWQ has yet to be validated with Asian Americans, the full 16-item version of this scale demonstrated good reliability (α = 0.81) in a sample of Asian American college students (50–52). Furthermore, whereas the full scale consists of two subscales tapping positively worded items and negatively worded items, respectively, the brief version includes five positively worded items only, resulting in a unidimensional scale (42). In the present study, the internal consistency was excellent for the overall sample (α = 0.96) and across ethnic subgroups (α = 0.94, 0.93, and 0.94 for East Asians, South Asians, and Southeast Asians, respectively).

#### Street race

To assess the likelihood that each participant believes they may be racially or ethnically misidentified, we modified a measure developed by López et al. (36) for Latinx Americans to assess “street race,” a perception of how other Americans on the street would perceive one's race (36). Participants were asked if they have ever been mistaken for a race/ethnicity other than their own and asked to indicate the top three racial or ethnic groups other than their own that they are frequently mistaken for. The response to the second question was manually coded for whether the participant reported being mistaken as Chinese or not. Chinese-identifying participants and non-Chinese participants who reported being mistaken as Chinese were coded as 1 (i.e., “Chinese or mistaken as Chinese”), and non-Chinese participants who reported never being mistaken as Chinese were coded as 0 (i.e., “neither Chinese nor mistaken as Chinese”)^1^.

#### Demographic information

Demographic covariates previously identified as significant predictors of discrimination, distress, and worry were included in our study. Age, gender (i.e., male, female), ethnicity, country of birth, years of residence in the United States, education level, and health insurance status were included. For the purposes of this study, we coded ethnicity into a categorical variable including three categories (1 = East Asian, 2 = South Asian, 3 = Southeast Asian). We created a binary variable of participants' nativity (1 = U.S. born, 0 = foreign born) using their country of birth. To control for socioeconomic status, we further coded participants' education level (i.e., less than college, some college or more) and health insurance status (i.e., uninsured, public insurance, private insurance).

### Analytic strategy

STATA v 17 was used for all analyses. As a preliminary step, we conducted one-way analysis of variance (ANOVA), chi-square tests to compare demographic characteristics across the ethnic subgroups (i.e., East Asian, South Asian, Southeast Asian). Then, we conducted *t-*tests and one-way ANOVAs to compare the means of COVID-19 stressors (i.e., pandemic stress, direct discrimination, vicarious discrimination) and psychological outcomes (i.e., distress, worry) by major categorical demographic characteristics (i.e., ethnic subgroup, street race, gender, nativity, education level, health insurance status). In order to assess the normality of data distribution, we examined its skewness and kurtosis and all the continuous except for pandemic stress were within ±3 and kurtosis within ±10 (53). For continuous variables (i.e., age), we examined their correlation with each of the study variables. We used Bonferroni *post-hoc* tests following significant ANOVAs for pairwise comparisons.

Finally, we conducted hierarchical regression analyses to examine whether experiences of discrimination were associated with negative psychological outcomes over and above the effects of pandemic stress. For each outcome, we entered demographic covariates (i.e., gender, age, nativity, education level, street race, health insurance status) in the first step (Model 1), pandemic stress in the second step (Model 2), and discrimination (i.e., direct, vicarious) in the final step (Model 3). To examine whether these relations vary by ethnicity, analyses were conducted separately for each ethnic subgroup (i.e., East Asian, South Asian, Southeast Asian).

## Results

### Demographic comparisons

Comparisons of demographic characteristics by ethnic subgroups are presented in Table 1. East Asian participants (M = 44.2, SD = 16.0) were significantly older than South Asian (M = 36.8, SD = 15.7) and Southeast Asian (M = 35.8, SD = 12.7) participants (F = 21.18, *p* < 0.001). We also found significant ethnic subgroup differences in nativity (χ2 = 10.15, *p* < 0.01), with more than half of East Asian participants (60.59%, *n* = 206) being foreign born and more than half of South Asian participants (54.33%, *n* = 69) being U.S. born. Nearly half of East Asian (48.85%, *n* = 166) and Southeast Asian (46.45%, *n* = 72) participants were residing in the Western U.S., whereas the largest geographic region represented by South Asian participants was the South (40.63%, *n* = 52; χ2 = 74.41, *p* < 0.001). The three groups did not differ significantly in their gender distribution, education level, and health insurance status; however, Southeast Asians had the highest proportion of participants with less than college education (24.18%), and East Asians had the highest proportion of those who had received some college education or more (83.53%). Whereas, the majority (84.80%; *n* = 259) of East Asian Americans either identified as Chinese or reported being mistaken as Chinese, and the majority (96.88%; *n* = 124) of South Asian Americans did not identify as Chinese or did not report being mistaken as Chinese, Southeast Asian Americans were more evenly split, with 53.55% (*n* = 83) reporting that they either identified as or had been mistaken as Chinese (χ2 = 226.06, *p* < 0.001).

Comparisons of study variables by major demographic characteristics are presented in Table 2. Ethnic subgroup differences in levels of pandemic stress were marginally significant (*p* = 0.057), with Southeast Asians reporting higher levels than East Asians (*p* = 0.07). The three ethnic subgroups varied significantly in their reported levels of direct discrimination (*p* < 0.01), but not vicarious discrimination (*p* = 0.37). *Post-hoc* analyses revealed that East Asian participants reported lower levels of direct discrimination than South Asian (*p* < 0.01) and Southeast Asian (*p* = 0.04) participants. East Asian participants also reported significantly lower levels of psychological distress than South Asian (*p* < 0.001) and Southeast Asian (*p* < 0.01) participants. Ethnic subgroup differences in levels of worry were marginally significant (*p* = 0.08), with Southeast Asians reporting higher levels than East Asians (*p* = 0.08). Furthermore, participants who identified as Chinese or were mistaken as Chinese reported significantly higher levels of distress (but not worry) than those who did not meet either criterion (*p* < 0.01).

**Table 2:** Comparisons of study variables by demographic characteristics.

	**Pandemic stress**		**Direct discrimination**		**Vicarious discrimination**		**Distress**		**Worry**	
	**M (SD)**	**t/F**	**M (SD)**	**t/F**	**M (SD)**	**t/F**	**M (SD)**	**t/F**	**M (SD)**	**t/F**
Ethnic group										
East Asian	8.8 (8.8)		1.5 (0.7)		2.0 (1.1)		20.2 (9.2)		14.7 (5.7)	
South Asian	8.7 (7.5)	2.87^†^	1.8 (1.0)	5.68**	2.1 (1.1)	1.01	24.4 (11.9)	12.62***	15.3 (6.1)	2.55^†^
Southeast Asian	10.7 (9.5)		1.7 (0.9)		2.2 (1.0)		23.9 (10.8)		16.0 (6.3)	
Gender										
Man	8.4 (7.2)	−1.96*	1.6 (0.8)	−0.10	2.0 (1.0)	−1.82*	20.4 (9.6)	−2.89*	13.8 (5.6)	−4.33***
Woman	9.8 (9.6)		1.6 (0.9)	−0.10	2.1 (1.1)		22.8 (10.7)		15.9 (6.0)	
Nativity										
U.S. born	10.0 (9.9)	−2.04*	1.7 (0.9)	−1.46	2.2 (1.1)	−3.03**	23.0 (10.6)	−2.85**	15.8 (6.0)	−2.73**
Foreign born	8.4 (7.2)		1.6 (0.8)		1.9 (1.0)		20.6 (10.0)		14.4 (5.8)	
Education										
Less than college	9.5 (8.5)	−0.32	1.7 (0.9)	−0.60	2.2 (1.1)	−1.73	24.8 (11.2)	−3.70***	16.3 (6.1)	−2.22*
Some college or more	9.2 (8.9)		1.6 (0.8)		2.0 (1.1)		21.2 (10.0)		14.8 (5.9)	
Health insurance status										
Uninsured	12.1 (9.8)	4.68**	2.0 (1.1)	5.51**	2.3 (1.2)	3.24*	27.2 (10.9)	8.81**	16.4 (6.3)	1.44
Public insurance	7.7 (6.9)		1.5 (0.8)		1.8 (0.9)		21.0 (10.0)		15.5 (6.2)	
Private insurance	9.3 (9.2)		1.6 (0.8)		2.1 (1.1)		21.4 (10.3)		14.8 (5.8)	
Street race										
Chinese or mistaken										
as Chinese	8.9 (8.7)	1.23	1.6 (0.8)	1.87^†^	2.1 (1.0)	−0.81	20.8 (9.5)	3.38**	14.9 (5.9)	1.03
Not Chinese and not										
mistaken as Chinese	9.8 (8.8)		1.7 (1.0)		2.0 (1.1)		23.6 (11.3)		15.4 (6.1)	

^†^p < 0.1, *p < 0.05, **p < 0.01, ***p < 0.001. Tests of group differences for ethnic groups and health insurance statuses show the F statistics. Tests of group differences for gender, nativity, and street race used the t statistics.

Women in our sample reported significantly higher levels of pandemic stress (*p* = 0.05), distress (*p* < 0.01), worry (*p* < 0.001), and vicarious discrimination (*p* = 0.03) than men, but no gender differences were found in experiences of direct discrimination (*p* = 0.59). U.S. born participants reported significantly higher levels of pandemic stress (*p* = 0.03), vicarious discrimination (*p* < 0.01), distress (*p* < 0.01), and worry (*p* < 0.01) than foreign born participants. We found no significant differences in levels of direct discrimination by nativity.

Participants who had received some college education of more were at significantly higher risk for distress (*p* < 0.001) and worry (*p* = 0.02) than those with less than college education, but the two groups did not differ in their pandemic stress (*p* = 0.75), direct discrimination (*p* = 0.55), and vicarious discrimination (*p* = 0.08). In general, uninsured participants were at the highest risk for pandemic stress, direct discrimination, vicarious discrimination, and distress compared to those with public and private insurance. Not included in Table 2, we found no significant differences in any of the study variables by geographic region.

Zero-order correlations revealed that age was negatively correlated with all study variables, including pandemic stress (r = −0.19, *p* < 0.001), direct discrimination (r = −0.30, *p* < 0.001), vicarious discrimination (r = −0.42, *p* < 0.001), distress (r = −0.45, *p* < 0.001), and worry (r = −0.37, *p* < 0.001) (see Table 3).

**Table 3:** Correlations among study variables.

**Variables**	***M* (*SD*)**	**1**	**2**	**3**	**4**	**5**	**6**
1. Age	40.63 (15.70)	-					
2. Pandemic stress	9.24 (8.79)	−0.19	-				
3. Direct discrimination	1.63 (0.85)	−0.30	0.24	-			
4. Vicarious discrimination	2.06 (1.06)	−0.42	0.35	0.74	-		
5. Distress	21.86 (10.33)	−0.46	0.30	0.54	0.51	-	
6. Worry	15.10 (5.95)	−0.37	0.23	0.35	0.41	0.69	-

M, Mean; SD, Standard Deviation, All correlations are significant at p < 0.001.

In sum, these results indicate that participants who are women, younger, born in the United States, and uninsured report higher levels of various COVID-19 stressors and negative psychological outcomes compared to those who are men, older, born outside of the United States, and have either private or public insurance. Our results also suggest that South Asian and Southeast Asian participants generally report higher levels of discrimination and negative psychological outcomes than East Asian participants, with ethnic subgroup differences being most pronounced in experiences of direct discrimination and distress.

### Hierarchical regressions

Prior to running the hierarchical regressions, we examined zero-order correlations among study variables (see Table 3). We found significant correlations among all study variables at the *p* < 0.001 significance level. Pandemic stress was moderately correlated with discrimination and psychological outcomes, with all coefficients falling below 0.40. Direct and vicarious discrimination showed moderate to strong correlations with negative psychological outcomes (r = 0.41–0.54) and strong correlations with one another (r = 0.74). Psychological distress and worry were also strongly correlated with each other (r = 0.69). While a correlation r greater 0.80 indicates the presence of multicollinearity (54), we examined the variance inflation factor (VIF) values for our regression models including all predictors for further investigation. All VIF values were estimated to be < 3.

Hierarchical ordinary least squares (OLS) regression analyses were conducted for each ethnic subgroup to examine the unique contributions of pandemic stress and discrimination after controlling for demographic covariates (see Tables 4–9). We assessed whether the ethnic subgroups varied in the predictors of psychological outcomes by comparing the confidence intervals of the standardized coefficients (β). If the 95% confidence intervals for a particular predictor did not overlap across groups, we concluded that there was a meaningful difference. We also note ethnic subgroup differences in the statistical significance of predictors (i.e., when a predictor significantly predicted outcomes in one subgroup but not in another).

**Table 4:** Hierarchical regressions for psychological distress: East Asian.

	**Model 1**		**Model 2**		**Model 3**	
	**Coef. (SE)**	**95% CI**	**Coef. (SE)**	**95% CI**	**Coef. (SE)**	**95% CI**
Woman (ref: man)	0.27 (0.95)	[−1.6, 2.13]	−0.16 (0.93)	[−2, 1.68]	−0.03 (0.84)	[−1.67, 1.62]
Age	−0.24 (0.03)***	[−0.3, −0.18]	−0.23 (0.03)***	[−0.29, −0.17]	−0.15 (0.03)***	[−0.21, −0.09]
Less than college (Ref: college and more)	−0.06 (1.29)	[−2.6, 2.47]	0.16 (1.26)	[−2.33, 2.64]	0.4 (1.13)	[−1.83, 2.63]
US Born (Ref: foreign Born)	1.43 (0.96)	[−0.46, 3.32]	1.26 (0.94)	[−0.59, 3.11]	1 (0.84)	[−0.65, 2.66]
Chinese or mistaken as Chinese (Ref: not Chinese and not mistaken)	−0.02 (0.95)	[−1.88, 1.85]	0.01 (0.93)	[−1.82, 1.84]	0.2 (0.84)	[−1.45, 1.84]
Health insurance status (ref: private insurance)						
Uninsured	3.75 (1.91)^†^	[−0.01, 7.51]	3.63 (1.87)	[−0.06, 7.31]	3.23 (1.67)^†^	[−0.06, 6.52]
Public insurance	0.28 (1.17)	[−2.02, 2.58]	0.17 (1.15)	[−2.08, 2.43]	0.34 (1.03)	[−1.68, 2.36]
Pandemic stress			0.2 (0.05)***	[0.1, 0.31]	0.11 (0.05)*	[0.01, 0.2]
Direct discrimination					5.34 (0.81)***	[3.75, 6.93]
Vicarious discrimination					0.19 (0.59)	[−0.97, 1.36]
R2	0.20		0.23		0.39	
ΔR2			0.04***		0.16***	

^†^p < 0.1, *p < 0.05, ***p < 0.001. Coef, Coefficient; SE, Standard Error; CI, Confidence Intervals.

**Table 5:** Hierarchical regressions for psychological distress: South Asian.

	**Model 1**		**Model 2**		**Model 3**	
	**Coef. (SE)**	**95% CI**	**Coef. (SE)**	**95% CI**	**Coef. (SE)**	**95% CI**
Woman (ref: man)	2.46 (2.01)	[−1.52, 6.43]	2.16 (1.88)	[−1.56, 5.87]	2.00 (1.67)	[−1.31, 5.31]
Age	−0.33 (0.07)***	[−0.46, −0.19]	−0.28 (0.07)***	[−0.41, −0.15]	−0.19 (0.06)**	[−0.31, −0.07]
Less than college (ref: college and more)	−2.13 (2.59)	[−7.25, 3]	−3.66 (2.44)	[−8.5, 1.18]	−2.66 (2.18)	[−6.97, 1.66]
US Born (ref: foreign born)	2.33 (2.07)	[−1.78, 6.44]	2.50 (1.94)	[−1.34, 6.33]	1.05 (1.76)	[−2.43, 4.53]
Health insurance status (ref: private insurance)						
Uninsured	5.55 (2.94)^†^	[−0.28, 11.38]	5.82 (2.75)*	[0.37, 11.27]	4.60 (2.47)^†^	[−0.29, 9.49]
Public insurance	3.41 (2.38)	[−1.31, 8.12]	5.00 (2.26)*	[0.53, 9.47]	4.65 (2)*	[0.68, 8.62]
Pandemic stress			0.52 (0.12)***	[0.27, 0.77]	0.35 (0.12)**	[0.11, 0.6]
Direct discrimination					2.71 (1.4)^†^	[−0.06, 5.49]
Vicarious discrimination					2.43 (1.5)	[−0.55, 5.4]
R2	0.21		0.27		0.34	
ΔR2			0.06***		0.07***	

^†^p < 0.1, *p < 0.05, **p < 0.01, ^***^p < 0.001. Coef, Coefficient; SE, Standard Error; CI, Confidence Intervals.

**Table 6:** Hierarchical regressions for psychological distress: Southeast Asian.

	**Model 1**		**Model 2**		**Model 3**	
	**Coef. (SE)**	**95% CI**	**Coef. (SE)**	**95% CI**	**Coef. (SE)**	**95% CI**
Woman (ref: man)	4.32 (1.64)**	[1.07, 7.56]	4.32 (1.56)**	[1.23, 7.42]	4.83 (1.4)**	[2.07, 7.6]
Age	−0.36 (0.07)***	[−0.5, −0.23]	−0.32 (0.07)***	[−0.45, −0.19]	−0.25 (0.06)***	[−0.37, −0.13]
Less than college (ref: college and more)	1.75 (1.91)	[−2.03, 5.54]	2.5 (1.83)	[−1.13, 6.13]	2.89 (1.64)^†^	[−0.35, 6.14]
US born (ref: foreign born)	−0.48 (1.65)	[−3.75, 2.79]	−1.19 (1.59)	[−4.33, 1.94]	−0.08 (1.42)	[−2.89, 2.74]
Chinese or mistaken as Chinese (Ref: not Chinese and not mistaken)	0.85 (4.74)	[−8.52, 10.22]	0.68 (4.52)	[−8.25, 9.61]	1.42 (4.03)	[−6.55, 9.39]
Health insurance status (ref: private insurance)						
Uninsured	5.48 (2.35)*	[0.83, 10.14]	3.58 (2.3)	[−0.96, 8.12]	2.61 (2.08)	[−1.51, 6.72]
Public insurance	4.89 (1.93)*	[1.08, 8.7]	5.46 (1.84)**	[1.82, 9.11]	4.95 (1.64)**	[1.71, 8.2]
Pandemic stress			0.31 (0.08)***	[0.15, 0.47]	0.2 (0.08)*	[0.05, 0.35]
Direct discrimination					4.11 (1.12)***	[1.89, 6.33]
Vicarious discrimination					0.71 (1.08)	[−1.42, 2.83]
R2	0.23		0.24		0.31	
ΔR2			0.02***		0.07***	

^†^p < 0.1, *p < 0.05, **p < 0.01, ***p < 0.001. Coef, Coefficient; SE, Standard Error; CI, Confidence Intervals.

**Table 7:** Hierarchical regressions for psychological worry: East Asian.

	**Model 1**		**Model 2**		**Model 3**	
	**Coef. (SE)**	**95% CI**	**Coef. (SE)**	**95% CI**	**Coef. (SE)**	**95% CI**
Woman (ref: man)	1.22 (0.6)*	[0.04, 2.39]	0.98 (0.59)†	[−0.19, 2.15]	0.97 (0.57)^†^	[−0.15, 2.1]
Age	−0.14 (0.02)***	[−0.18, −0.1]	−0.13 (0.02)***	[−0.17, −0.1]	−0.1 (0.02)***	[−0.14, −0.06]
Less than college (ref: college and more)	−0.18 (0.81)	[−1.78, 1.42]	−0.06 (0.8)	[−1.64, 1.52]	−0.07 (0.78)	[−1.6, 1.45]
US born (ref: foreign born)	1 (0.61)	[−0.2, 2.19]	0.9 (0.6)	[−0.27, 2.08]	0.79 (0.58)	[−0.34, 1.93]
Chinese or mistaken as Chinese (Ref: not Chinese and not mistaken)	0.05 (0.6)	[−1.13, 1.23]	0.07 (0.59)	[−1.1, 1.23]	0.02 (0.57)	[−1.1, 1.15]
Health insurance status (ref: private insurance)						
Uninsured	1.12 (1.21)	[−1.26, 3.49]	1.05 (1.19)	[−1.29, 3.39]	0.98 (1.15)	[−1.27, 3.24]
Public insurance	1.54 (0.74)*	[0.08, 2.99]	1.48 (0.73)*	[0.04, 2.91]	1.5 (0.7)*	[0.12, 2.88]
Pandemic stress			0.11 (0.03)**	[0.05, 0.18]	0.07 (0.03)*	[0, 0.13]
Direct discrimination					1.27 (0.55)*	[0.18, 2.36]
Vicarious discrimination					0.8 (0.4)^†^	[0, 1.59]
R2	0.20		0.23		0.39	
ΔR2			0.04***		0.16***	

^†^p < 0.1, *p < 0.05, **p < 0.01, ***p < 0.001. Coef, Coefficient; SE, Standard Error; CI, Confidence Intervals.

**Table 8:** Hierarchical regressions for psychological worry: South Asian.

	**Model 1**		**Model 2**		**Model 3**	
	**Coef. (SE)**	**95% CI**	**Coef. (SE)**	**95% CI**	**Coef. (SE)**	**95% CI**
Woman (ref: man)	2.2 (1.04)*	[0.13, 4.26]	2.08 (1.01)*	[0.08, 4.07]	2.03 (0.97)*	[0.1, 3.95]
Age	−0.13 (0.04)***	[−0.2, −0.06]	−0.11 (0.04)**	[−0.18, −0.04]	−0.08 (0.04)*	[−0.15, −0.01]
Less than college (ref: college and more)	−0.26 (1.35)	[−2.92, 2.41]	−0.86 (1.31)	[−3.46, 1.74]	−0.51 (1.27)	[−3.02, 2]
US born (ref: foreign born)	1 (1.08)	[−1.14, 3.14]	1.06 (1.04)	[−1, 3.13]	0.57 (1.02)	[−1.46, 2.59]
Health insurance status (ref: private insurance)						
Uninsured	2.28 (1.53)	[−0.75, 5.31]	2.39 (1.48)	[−0.54, 5.31]	1.95 (1.44)	[−0.89, 4.8]
Public insurance	2.63 (1.24)*	[0.17, 5.08]	3.26 (1.21)**	[0.85, 5.66]	3.14 (1.16)**	[0.83, 5.44]
Pandemic stress			0.21 (0.07)**	[0.07, 0.34]	0.15 (0.07)*	[0.01, 0.29]
Direct discrimination					0.98 (0.82)	[−0.64, 2.59]
Vicarious discrimination					0.81 (0.87)	[−0.92, 2.54]
R2	0.25		0.35		0.50	
ΔR2			0.10***		0.15***	

*p < 0.05, **p < 0.01, ***p < 0.001. Coef, Coefficient; SE, Standard Error; CI, Confidence Intervals.

**Table 9:** Hierarchical regressions for psychological worry: Southeast Asian.

	**Model 1**		**Model 2**		**Model 3**	
	**Coef. (SE)**	**95% CI**	**Coef. (SE)**	**95% CI**	**Coef. (SE)**	**95% CI**
Woman (ref: man)	2.00 (1.03)^†^	[−0.05, 4.04]	2 (1.03)^†^	[−0.03, 4.03]	2.06 (0.99)*	[0.1, 4.03]
Age	−0.22 (0.04)***	[−0.3, −0.13]	−0.21 (0.04)***	[−0.29, −0.12]	−0.16 (0.04)**	[−0.25, −0.07]
Less than college (ref: college and more)	−0.15 (1.21)	[−2.53, 2.24]	0.06 (1.21)	[−2.32, 2.45]	0.4 (1.17)	[−1.9, 2.71]
US born (ref: foreign born)	−0.64 (1.04)	[−2.7, 1.42]	−0.84 (1.04)	[−2.9, 1.22]	−0.51 (1.01)	[−2.51, 1.49]
Chinese or mistaken as Chinese (Ref: not Chinese and not mistaken)	2.12 (2.99)	[−3.78, 8.02]	2.08 (2.97)	[−3.79, 7.94]	2.01 (2.86)	[−3.65, 7.67]
Health insurance status (ref: private insurance)						
Uninsured	0.01 (1.48)	[−2.92, 2.94]	−0.53 (1.51)	[−3.51, 2.46]	−0.45 (1.48)	[−3.37, 2.47]
Public insurance	2.62 (1.21)*	[0.23, 5.02]	2.79 (1.21)*	[0.4, 5.18]	2.65 (1.17)*	[0.35, 4.96]
Pandemic stress			0.09 (0.05)^†^	[−0.02, 0.19]	0.02 (0.05)	[−0.08, 0.13]
Direct discrimination					0.4 (0.8)	[−1.17, 1.98]
Vicarious discrimination					1.57 (0.76)*	[0.06, 3.08]
R2	0.31		0.38		0.52	
ΔR2			0.07***		0.14***	

^†^p < 0.1, *p < 0.05, **p < 0.01, ***p < 0.001. Coef, Coefficient; SE, Standard Error; CI, Confidence Intervals.

Regarding demographic variables, younger age was significantly associated with increases in distress and worry across all three groups and all three steps, including the final model accounting for pandemic stress and discrimination (Model 3). Being female was a stronger predictor of distress in Southeast Asians than East Asians. Whereas gender showed nearly null associations with psychological distress among East Asians across all three steps (b = −0.025, *p* = 0.98 in Model 3), being female was significantly related to distress (b = 4.83, *p* < 0.01) over and above the contributions of pandemic stress and discrimination among Southeast Asians. It is also worth noting that although female gender was not significantly associated with distress among South Asians, it was a significant predictor of worry across all three steps (b = 2.03, *p* = 0.04).

In general, health insurance status was a more robust predictor of negative psychological outcomes for South Asian and Southeast Asian participants than for East Asian participants. Although the confidence intervals overlapped across ethnic subgroups, having public insurance (as opposed to private insurance) was significantly related to higher distress among South Asian and Southeast Asian participants (b = 4.65, *p* = 0.02 for South Asians, b = 5.00, *p* < 0.01 for Southeast Asians, Model 3), whereas insurance status did not predict distress among East Asians (b = 0.34, *p* = 0.74). Being uninsured was also generally associated with worse outcomes across ethnic subgroups, but none of these associations were significant in any of the final models. Nativity, education level, and Chinese street race (i.e., whether one identifies, or reports being mistaken as Chinese) were not significantly related with distress and worry for any of the ethnic subgroups. For South Asians, the sample size for those reporting being mistaken for Chinese was very small (*n* = 4), thus this variable was omitted from the regression models.

Pandemic stress was entered in the second step (Model 2), and the change in R2 was significant across ethnic subgroups for both psychological outcomes. Direct and vicarious discrimination were simultaneously entered in the third step (Model 3), and the R2 was significant across ethnic subgroups for both outcomes. After accounting for discrimination, the coefficient for pandemic stress decreased slightly but remained statistically significant in predicting distress for all three ethnic subgroups and in predicting worry among South Asians and East Asians. Thus, pandemic stress significantly contributed to worse psychological outcomes in our sample after controlling for demographic variables, and in turn, experiences of direct and vicarious discrimination significantly contributed to these outcomes over and above the effects of demographic variables and pandemic stress.

In the final model (Model 3), we found several notable ethnic subgroup differences regarding the contributions of pandemic stress, direct discrimination, and vicarious discrimination. Pandemic stress was a significant predictor of distress across ethnic subgroups and of worry among South Asians and East Asians, with South Asians showing the largest coefficients for both outcomes (b = 0.35, *p* < 0.01 for distress; b = 0.15, *p* = 0.04 for worry). On the other hand, neither direct nor vicarious discrimination significantly predicted distress or worry among South Asians, except for the marginally significant association between direct discrimination and distress (b = 2.71, *p* = 0.06).

Experiences of direct discrimination were significantly and positively associated with both distress (b = 5.34, *p* < 0.001) and worry (b = 1.27, *p* = 0.02) among East Asians, whereas it significantly predicted distress (b = 4.11, *p* < 0.001) but not worry (b = 0.40, *p* = 0.61) among Southeast Asians. Thus, despite reporting lower levels of both direct discrimination and negative psychological outcomes than South Asians and Southeast Asians, East Asian participants demonstrated the strongest associations between these variables.

Vicarious discrimination was a poor predictor of distress across all three groups, but it was significantly related with increases in worry Southeast Asian (b = 1.57, *p* = 0.04) participants, though not among East Asian (b = 0.80, *p* = 0.06) and South Asian participants (b = 0.81, *p* = 0.35). As mentioned above, whereas direct (but not vicarious) discrimination significantly contributed to distress among Southeast Asians, vicarious (but not direct) discrimination significantly contributed to their worry.

## Discussion

### Primary findings

Despite political references blaming China (and those associated with China) for the spread of the COVID-19 virus that developed into a worldwide pandemic, much of the public discourse in the U.S. about COVID-19-related discrimination and hate have referred to the targeted group at the aggregated racial level (e.g., anti-Asian hate) and sometimes even extending to Pacific Islanders (e.g., “#StopAAPIHate”). Consequently, a significant portion of social science research on the mental health impact of COVID-19 has also been conducted at the racial group level without further disaggregation, which has the potential to gloss over important differences across Asian ethnic subgroups with respect to sociodemographic factors as well as their racialization. By disaggregating Asian Americans by ethnic subgroup as well as socioeconomic class, gender, nativity, geographic regions, and age, the current study highlights the vastly different experiences and impacts of COVID-19 within the Asian American population.

Descriptive analyses with the total sample found that survey respondents who are women, younger, and U.S. born reported greater stress due to life events during COVID-19, greater vicarious discrimination, greater psychological distress, and more worry compared to those who are men, older, and foreign-born. These findings are largely consistent with the literature. For example, the finding that Asian American women experience greater pandemic stress, distress, and worry than men in our study is consistent with women being at higher risk for mental health during the pandemic in the general population (55). Comparing K-10 distress scores and the Penn State Worry Scale scores of our sample against published norms indicate that people with mean scores similar to our sample had a moderate to high likelihood (48.5–69.4%) of meeting the DSM-IV criteria for any mental disorder in the past 12 months (48, 56), although the mean level of worry reported by our sample was at the subclinical level (57, 58). Although non-diagnostic, the level of distress expressed by Asian Americans in this study adds to the growing evidence of the mental health burden in context of the COVID-19 pandemic.

With respect to racial discrimination, a pre-COVID study with Asian American college students had found inconsistent patterns of differences with respect to gender and nativity status on indices of subtle and blatant racism (59). With regard to age, we speculate that during the pandemic, younger Asian Americans may have been more connected than older Asian Americans to social media and other online platforms, which may have contributed to their increased awareness of reports of anti-Asian racial sentiments and hate incidents. It is also possible that the U.S. born Asian Americans were more likely to have been racialized (e.g., through U.S. schools, media, and neighborhoods) as visible minorities and thus more attuned to various forms of anti-Asian discrimination (60).

There were also some notable ethnic subgroup differences. Our analysis suggested that South and Southeast Asian Americans reported higher levels of psychological distress than did East Asian Americans, a pattern that was consistent with some but not all previous studies. For example, in an analysis of survey data collected from Chinese American and South Asian American residents of Chicago (29), South Asian Americans reported significantly more depressive symptoms than did Chinese Americans during COVID-19. Another study of Asian Canadians had also found that mental health symptoms increased more among South Asian Canadians (along with Black and Muslim Canadians) compared to East Asian, Southeast Asian, and White Canadians (61). Huynh et al. (26) had also reported that Southeast Asian Americans and East Asian American adults whose ethnicity were not Chinese reported more anxiety and depression during COVID-19 than those who identified as Chinese (26).

On its face, these results from these ethnic subgroup comparisons seem counter intuitive. With politicians and pundits blaming the Chinese for the pandemic, why did South Asians in the U.S. and Canada report *more* racial discrimination and psychological distress during COVID-19 than their East Asian and Chinese counterparts? One possibility, as suggested by Lozano et al. (21), is that Chinese Americans tend to live in more ethnically concentrated neighborhoods where they may be more protected from racial discrimination and distress. It is also possible that relatively recent experiences with post-9/11 xenophobia and racism may have primed South Asians to report more racial discrimination and distress (15, 19). Moreover, there is some data to suggest that Asian ethnic groups experienced a differential disease burden of COVID-19, especially during the initial months of the pandemic (23). Further research is needed to better understand how the particular racialized experiences as well as COVID-19 disease burden of each Asian ethnic community within the U.S. shape their perceptions of, and responses to, COVID-19 anti-Asian racism.

A novel aspect of our study was that in addition to Asian ethnicity, we also assessed street race to examine to what extent being Chinese or being mistaken as Chinese might be associated with discrimination and distress. However, our analyses revealed that Chinese street race was not significantly associated with direct or vicarious discrimination. In fact, Asian Americans who were neither Chinese nor had been mistaken as Chinese reported *more* psychological distress than those who were Chinese themselves. When this variable was entered into regression analyses along with other demographic predictors, Chinese street race was not a significant predictor of psychological distress or worry for any ethnic subgroups, including for Southeast Asian Americans, about half of whom had been mistaken as Chinese. Thus, at least in this sample, the prospect of being targeted by Sinophobic discrimination (1) did not explain variability in mental health outcomes. It is possible that by the time of our data collection in December 2020, in context of a widespread reference to #StopAAPIHate and the racial animus characterized as anti-Asian rather than specifically anti-Chinese (2, 3), any potential impact of being Chinese or being mistaken as Chinese was overwhelmed by the lived experiences of direct or vicarious attacks against Asians.

Our results also highlight some notable differences in the factors associated with poorer mental health across three major Asian American ethnic subgroups. For example, although East Asian Americans had reported less discrimination and distress in the group comparisons, regression analyses revealed that East Asian Americans who were younger, had experienced greater pandemic life stress, and were directly targeted by anti-Asian racism were the most distressed within this group. Southeast Asian Americans' levels of distress were also associated with younger age, stressful life events, and direct discrimination, but being female and being on public health insurance conferred additional risk in this group. In contrast, among South Asian Americans, neither direct nor vicarious discrimination were significantly associated with their distress; only younger age, health insurance status (i.e., public insurance), and stressful life events was associated with risk for greater psychological distress. The patterns of predictors of worry for ethnic subgroups largely resemble those for psychological distress, notably with the finding that neither direct nor vicarious discrimination were significant risk factors for South Asian Americans' worry. There may not be a simple explanation for these differential patterns of psychological distress for the ethnic subgroups, as they likely reflect population characteristics uniquely shaped by different immigration and racialization history. Nevertheless, these findings reinforce the critical importance of disaggregating COVID-19 data on Asian Americans.

Separate regression analyses for each ethnic subgroup revealed some common predictors. Across all three ethnic subgroups, age remained a significant predictor for both psychological distress and worry for all three ethnic subgroups, even when the effects of other demographic factors and stressors were taken into account. This finding that younger Asian American adults reported more mental health problems than older Asian American adults during the first year of COVID-19 pandemic adds to the cumulative evidence of a widespread mental health crisis among young adults (56, 57).

### Limitations and conclusion

The present findings must be interpreted with caution due to several limitations. First, the current study's data represent a convenience sample collected through a commercial online survey company and administered only in the English language. Thus, our findings may not necessarily reflect the general Asian American population. For example, in 2019, 72% of all Asians residing in the U.S. were proficient in English, whereas only 57% of foreign-born Asians were English proficient (58). Thus, the current study's participants likely reflect a more acculturated sample who could access online English-language surveys, which may reflect a more technologically savvy, college-educated sample with higher socioeconomic status than Asian American population at large. Our sampling criteria was purposefully inclusive, and the geographic distribution as well as the largest ethnic groups in this sample roughly mirror the proportion of various Asian ethnic groups in the U.S. population and regions. However, by including any adult who identified as Asian American, some of the ethnic groups (e.g., Pakistani Americans) were too small to be able to carry out ethnic-specific analyses. We were able to disaggregate the data by broad ethnic subgroups (East Asian, Southeast Asian, and Southeast Asian) to reveal notable differences, but we must be mindful that critical points of heterogeneity within each subgroup are nevertheless elided. For example, the largest three ethnic groups in our study (Chinese, Filipino, and Indian) constituted a sizable proportion of each subethnic group (Chinese were 61% of the East Asian sample, Filipinos were 45% of the Southeast Asian sample, and Indians were 63% of the South Asian sample), and results must be interpreted with this caveat in mind. Our study also did not sample Pacific Islanders who are often grouped together with Asian Americans but have vastly different historical and contemporary experiences. Furthermore, our study is based on cross-sectional data collected in December 2020, which reflects a particular point in the COVID-19 pandemic in the United States (~9 months into the pandemic, before vaccines became widely available, and prior to the precipitous spike in violent assaults against Asian American women and elders in 2021). Prior data from the #StopAAPIHate online reporting portal indicate that the number of COVID-19 related anti-Asian racial discrimination and hate incidents reported in 2021 increased since 2020 (2), indicating that anti-Asian racism will likely continue to impact the population even as the COVID-19 health crisis recedes.

Despite these caveats, our study makes novel contributions to the growing literature documenting the mental health costs of COVID-19 among Asian Americans. The findings of differential sets of risk factors for mental health outcomes by major ethnic subgroups underscore the importance of disaggregating Asian American data and to attend to the intersecting systems of oppression that shape the everyday lives of this diverse population. For example, we found that South Asian and Southeast Asian Americans perceived more COVID-19 related anti-Asian discrimination than did East Asian Americans, regardless of whether they had been mistaken on the street as Chinese, whereas the adverse psychological impacts of direct discrimination were strongest for East Asian Americans. Although Chinese “street race” in our study was not associated with higher reports of racial discrimination, the construct of street race may still be associated with mental health outcomes outside of the pandemic-fueled racial context (18). Additional theoretical and empirical work on street race among Asian American population may yield further insights into the experiences of racial and ethnic identity. Additional methods for assessing street race, such as having third-party perceptions of an Asian American individual's race and ethnicity based on facial images (62), may be able to supplement self-reports of being mistaken for another race or ethnicity. Furthermore, we found that being a woman and being on public health insurance was associated with worse psychological outcomes for South and Southeast Asians, but not for East Asian Americans. Given these findings, practitioners and policy makers must attend to a more nuanced understanding of how racism, sexism, and classism intersect to shape the lived experiences and wellbeing of Asian Americans. Moreover, research is needed to understand how colorism, Islamophobia, and the unique historical context of “Brown Asian Americans” inform how Southeast Asian and South Asian groups have experienced what has been commonly understood as anti-Chinese and anti-East Asian racism during COVID-19, in ways that may be quite different from how East Asian Americans have experienced racism (13). These intersectionalities matter in how civil society responds in more inclusive ways to the diversity of Asian American experiences with xenophobia and racial hostility exacerbated by the global pandemic.

## Data availability statement

The raw data supporting the conclusion of this article is available upon request to the corresponding author/s.

## Ethics statement

The studies involving human participants were reviewed and approved by NYU Institutional Review Board. The patients/participants provided their online informed consent to participate in this study.

## Author contributions

SO, AP, and CSL: conceptualization, methodology, data curation, data analysis, and writing—original draft/review and editing. DC: conceptualization, funding acquisition, writing, review, and editing. NY: methodology, data management, data analysis, data curation, and writing. All authors contributed to the article and approved the submitted version.

## Funding

This work was supported by the NYU Silver's Office for Research Seed Fund.

## Conflict of interest

The authors declare that the research was conducted in the absence of any commercial or financial relationships that could be construed as a potential conflict of interest. The reviewer YY declared a shared affiliation with the authors to the handling editor at the time of review.

## Publisher's note

All claims expressed in this article are solely those of the authors and do not necessarily represent those of their affiliated organizations, or those of the publisher, the editors and the reviewers. Any product that may be evaluated in this article, or claim that may be made by its manufacturer, is not guaranteed or endorsed by the publisher.

## Footnotes

^1^Because the study was concerned with Sinophobia and anti-Chinese racism as potentially more harmful than general anti-Asian racism, we grouped all who identified as Chinese regardless of their street race. T-tests comparing the K10 scores and PSWQ scores between those Chinese origin and not mistaken as non-Chinese (*n* = 137) vs. participants of Chinese origin and mistaken as non-Chinese (*n* = 56) revealed that the two did not differ significantly on measures of psychological distress or worry t(191) = 0.82, t(191) = 0.87, respectively.
